# Supplementary material for: Aschoff’s rule on circadian rhythms orchestrated by blue light sensor CRY2 and clock component PRR9
Source: Nat Commun. 2022 Oct 5;13:5869. doi: 10.1038/s41467-022-33568-3 (PMC9535003; doi:10.1038/s41467-022-33568-3)
Supplement: Supplementary file 1 — Supplementary Information [file 41467_2022_33568_MOESM1_ESM.pdf]

Aschoff's Rules on Circadian Rhythms Orchestrated by Blue Light Sensor CRY2 and Clock Component PRR9

Yuqing He,[1,2] Yingjun Yu,[1,2] Xiling Wang,[1,2] Yumei Qin,[1,2] Chen Su,[1,2] and Lei Wang,[1,2,*]

[1] Key laboratory of Plant Molecular Physiology, Institute of Botany, Chinese Academy of Sciences, Beijing, 10093, China

[2] University of Chinese Academy of Sciences, Beijing, 100049, China

*email: wanglei@ibcas.ac.cn

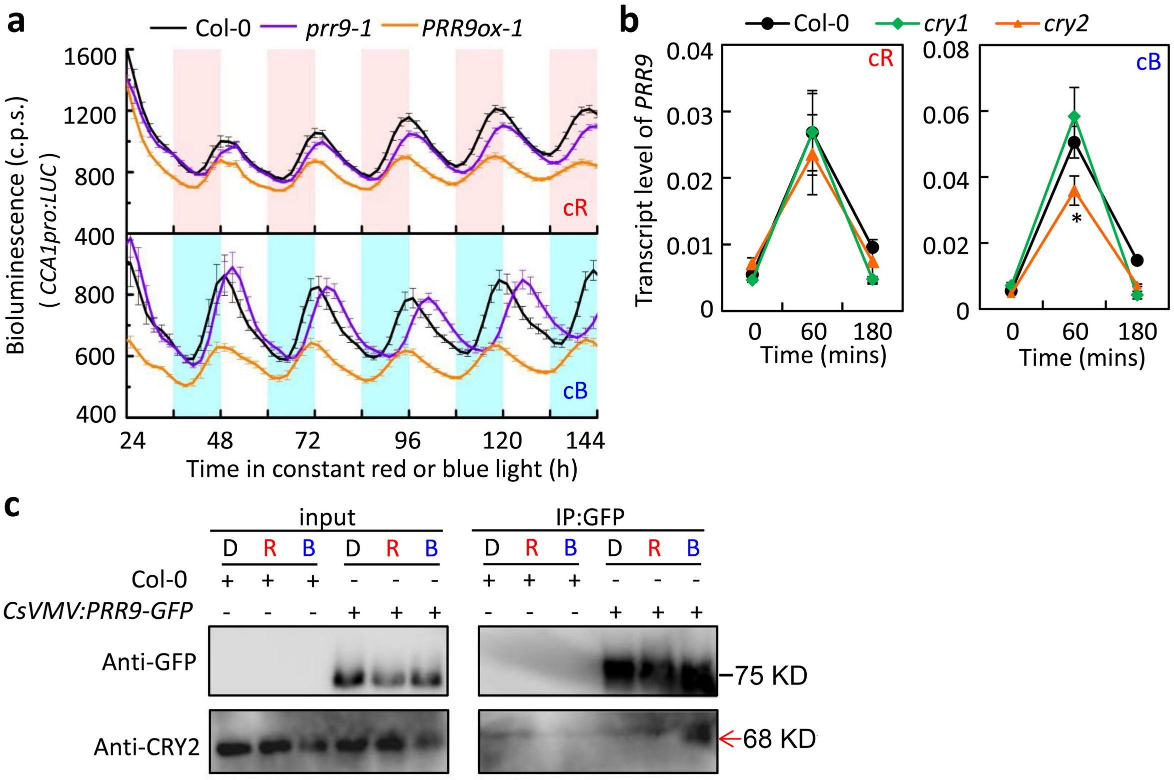

Supplementary Fig. 1: PRR9 is involved into blue light input.

a Bioluminescence trace of *CCA1pro:LUC* in Col-0, *prr9-1* and *PRR9ox-1* plants in constant 40 μmol m^{-2} s^{-1} red light (cR) (n=20, 20, 19 respectively) or blue light (cB) (n=20, 20, 20 respectively) conditions. Representative data from three biological repeats. Data represent mean ± s.e.m. **b** Expression profile of *PRR9* in Col-0, *cry1* or *cry2* mutant determined by RT-qPCR. Seedlings are grown in LD condition for 10 days, then transferred to red or blue light (40 μmol m^{-2} s^{-1}) for indicated durations. Data are mean ± s.d., n=3, technical repeats.

(*p < 0.05, Student's *t*-test) **c** Co-IP assay showing CRY2 interacted with PRR9 under blue light in *Arabidopsis.* 7-day-old Col-0 and *CsVMV:PRR9-GFP* seedlings were exposed to dark, red (R) and blue light (B) (10 μmol m^{-2} s^{-1}) respectively for 10 mins. Red arrow indicates endogenous CRY2 recognized by CRY2 antibody. Representative data from three independent biological repeats.

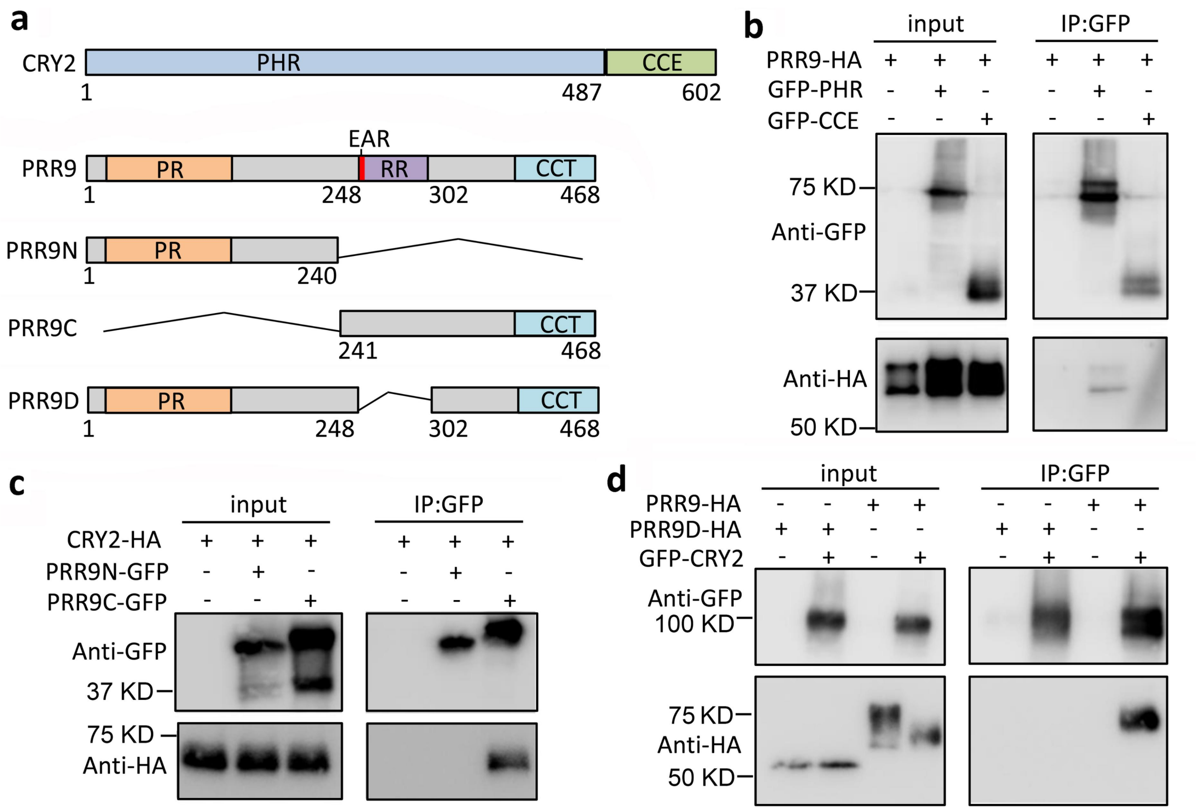

Supplementary Fig. 2: PHR domain of CRY2 is required for interacting with C-terminal of PRR9.

a Schematic diagram showing domain structure of CRY2, PRR9 and their truncated proteins used for CoIP assays. Numbers indicated the amino acid position. **b** PHR domain of CRY2 interacted with PRR9 in *N. benthamiana* leaves by Co-IP assay The IPed (PHR or CCE) and co-IPed signals (PRR9) were detected in immunoblots probed with GFP and HA antibodies, respectively. **c** PRR9C-GFP interacted with CRY2 in *N. benthamiana* leaves by Co-IP assay The IP (PRR9N or PRR9C) and co-IP signals (CRY2) were detected in immunoblots with GFP and HA antibodies, respectively. **d** Co-IP assay showing PRR9D can not interact with CRY2 in *N. benthamiana* leaves. The IP (CRY2) and co-IP signals (PRR9 or PRR9D) were detected in immunoblots with GFP and HA antibodies, respectively. For figure (b-d), similar results were observed from three independent biological repeats.

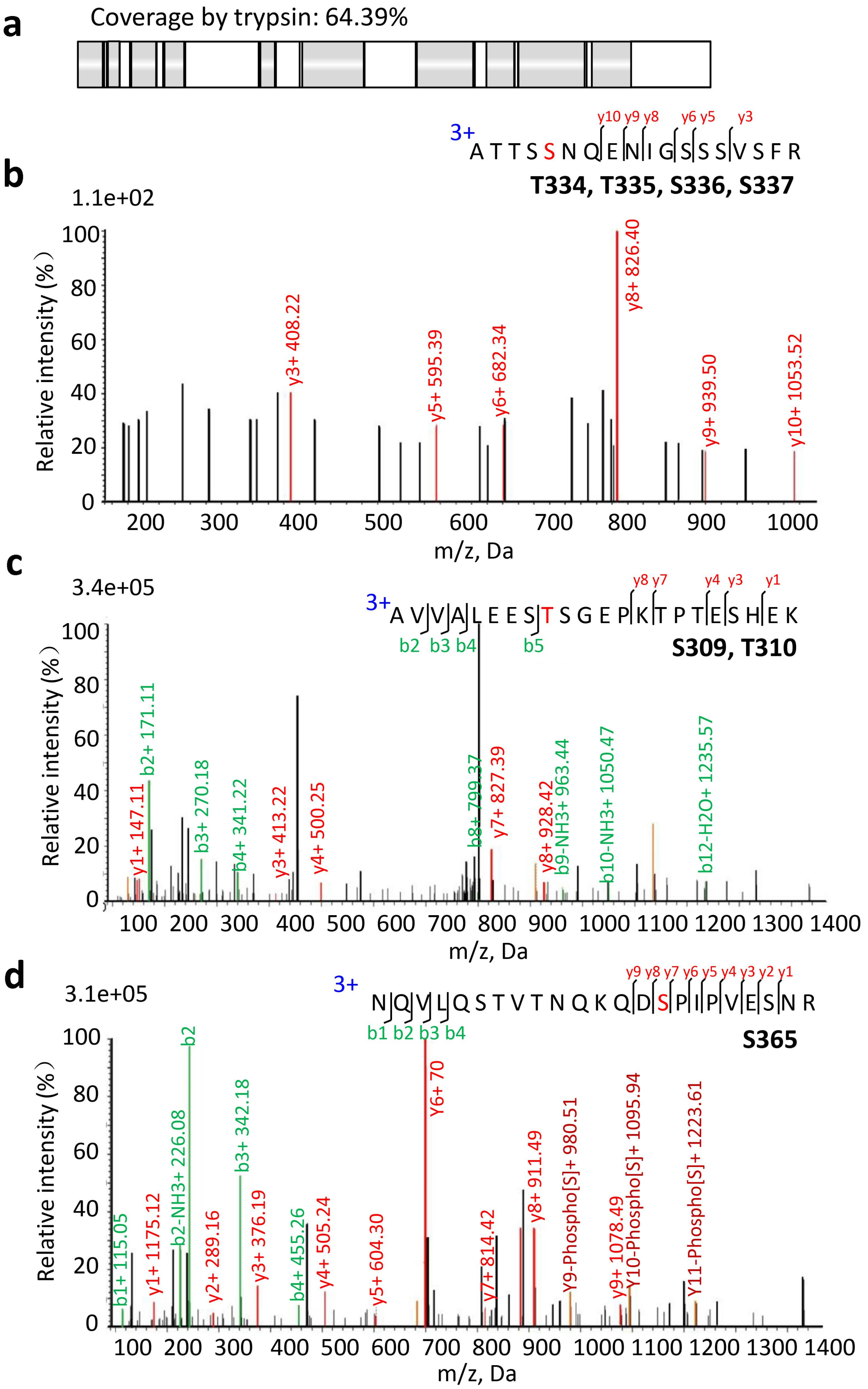

Supplementary Fig. 3: PRR9 peptides coverage and higher-energy collisional dissociation spectra of the phosphopeptides identified by mass spectrometry.

a Mass spectrometry coverage of PRR9 digested by trypsin from three biological replicates.

Regions in gray were identified by mass spectrometry while blank regions were not detected. **b-d** Mass spectrogram of three representative phosphopeptides ATTSSNQENIGSSSVSFR (b), AVVALEESTSGEPKTPTESHEK (c) and NQVLQSTVTNQKQDSPIPVESNR (d). Black bold characters are putative phosphosites on the phosphopeptide.

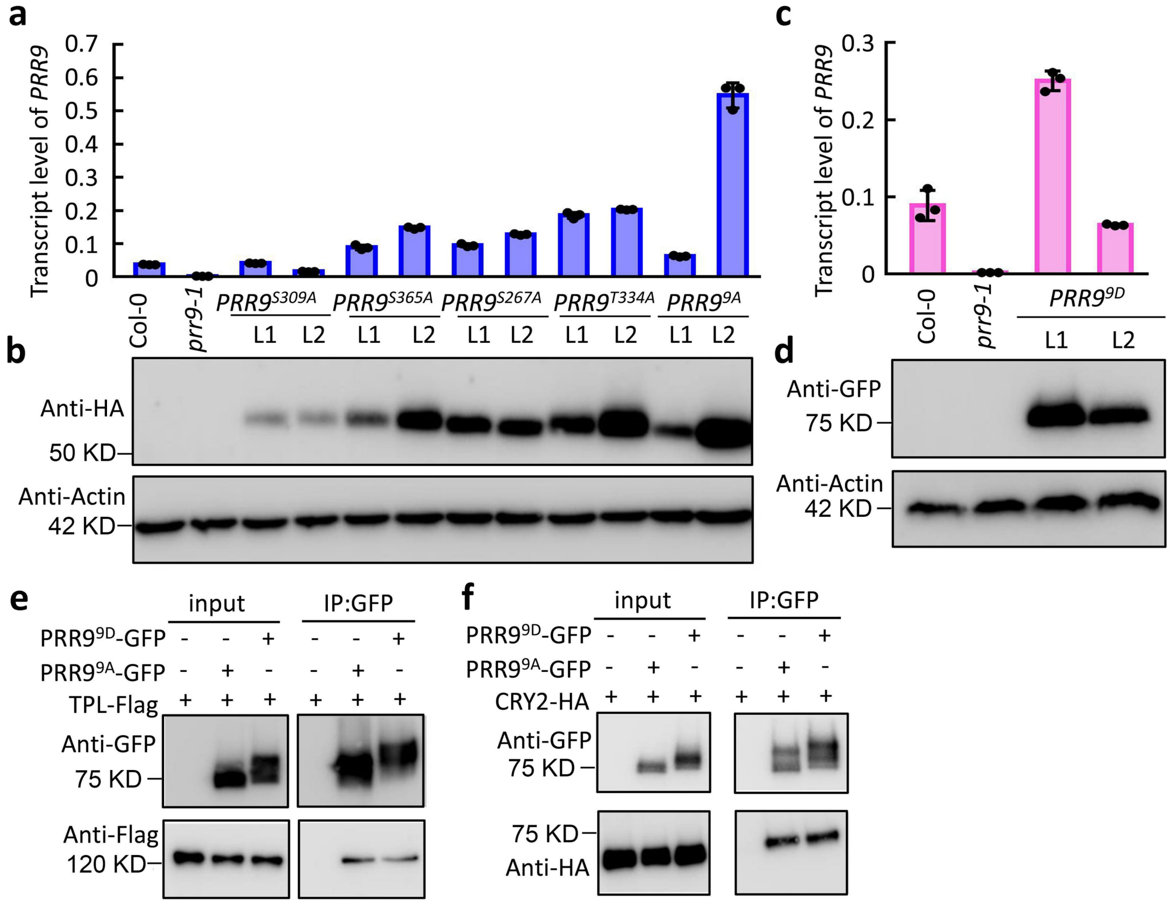

Supplementary Fig. 4: Characterization of PRR9 phospho-mutants.

a, b *PRR9* transcript levels at ZT1 (a) and protein levels at ZT5 (b) from Col-0, *prr9-1* and respective two independent lines of PRR9 phosphorylation-point mutation variants driven by native promoter tagged with HA in *prr9-1* background were detected. Data in (a) are mean ± s.d., n=3, technical repeats. Actin was used as a protein loading control in (b). **c, d** *PRR9* transcript levels at ZT1 (c) and protein levels at ZT5 (d) from Col-0, *prr9-1* and *PRR9:PRR9*^{9D}*-GFP* in *prr9-1* background (*PRR9*^{9D}). Data in (c) are mean ± s.d., n=3, technical repeats. Actin was used as a loading control in (d). **e, f** Co-IP assay showing phosphorylation of PRR9 has no effect on its interaction with TPL (e) and CRY2 (h) in planta from three biological repeats.

a

Spectral counts for PRR9 interacting proteins			
	Name	Repeat 1	Repeat 2
Bait:	PRR9	37	11
	PPK1	12	4
	PPK2	10	6
	PPK3	0	4
	PPK4	0	3

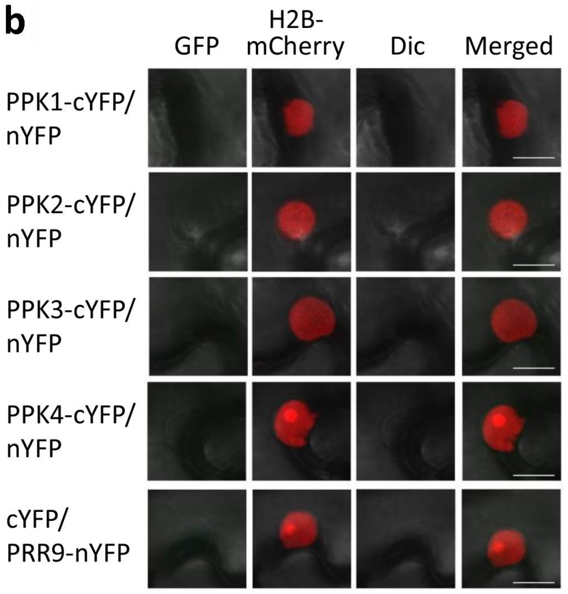

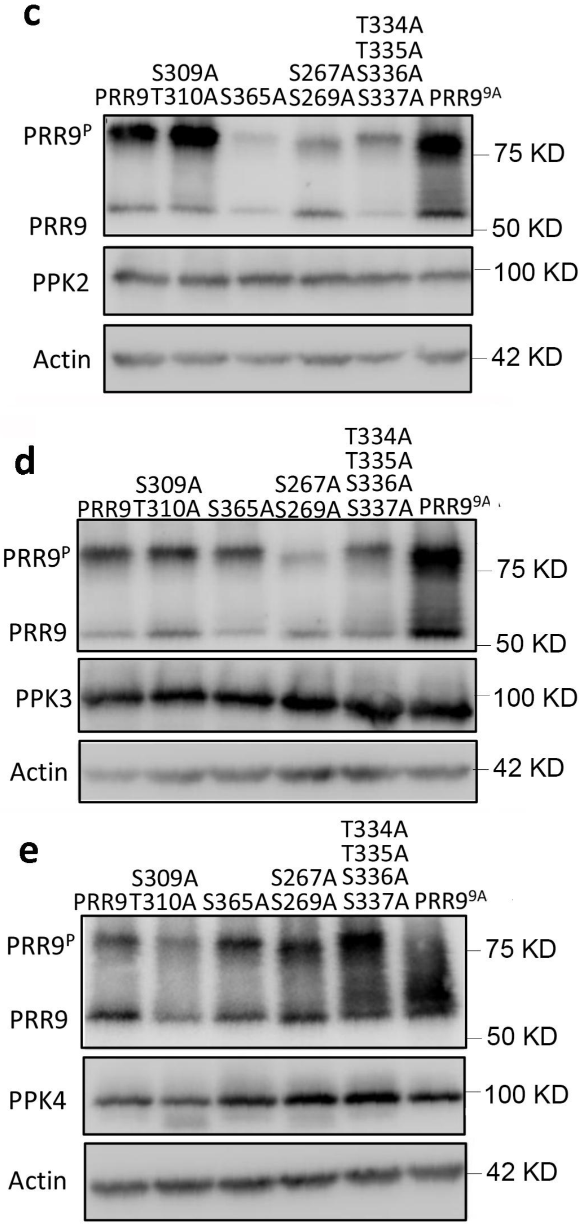

Supplementary Fig. 5: PPKs interact with PRR9 *in vitro and in vivo*.

a PRR9-interacting proteins identified by IP-MS analysis. Spectral counts were from two biological replicates. **b** Negative controls for BiFC assay. PPKs-cYFP and cYFP were individually co-expressed with nYFP or PRR9-nYFP as indicated in *N. benthamiana* leaves. H2B-mCherry as a nucleus marker. Bars, 20 μm. (n=12, 12, 12, 12 cells/2 leaves) **c-e** Immunoblot showing GFP tagged-PPK2 (c), PPK3 (d) and PPK4 (e) catalyzed phosphorylation of PRR9 and series of PRR9 mutants fused with HA tag. Total protein lysates were analyzed by immunoblot with GFP and HA antibodies, respectively. Actin was

used as a loading control. Similar results were observed from three independent biological repeats.

Supplementary Table 1: Circadian period length of Col-0, *prr9-1*, *cry2* and *cry2 prr9-1* under constant blue light with different light quantity.

	Period (h) ±S.E.M			
**cB (μmol m^{-2} s^{-1})**	**Col-0**	***prr9-1***	***cry2***	***cry2 prr9-1***
2.5	24.33±0.11	26.55±0.13	25.77±0.24	26.22±0.18
10	24.41±0.07	26.86±0.07	26.02±0.14	26.83±0.11
20	23.62±0.07	25.65±0.14	24.45±0.14	25.39±0.12

Supplementary Table 2: Circadian period length of Col-0, *cry2, PRR9ox-1* and *cry2 PRR9ox-1* under constant blue light with different light quantity.

	Period (h) ±S.E.M			
**cB (μmol m^{-2} s^{-1})**	**Col-0**	***cry2***	***PRR9ox-1***	***cry2 PRR9ox-1***
2.5	24.54±0.11	25.24±0.24	24.30±0.21	23.80±0.12
10	24.43±0.10	24.97±0.19	23.96±0.08	23.81±0.09
20	23.90±0.05	24.42±0.08	23.68±0.08	23.57±0.13

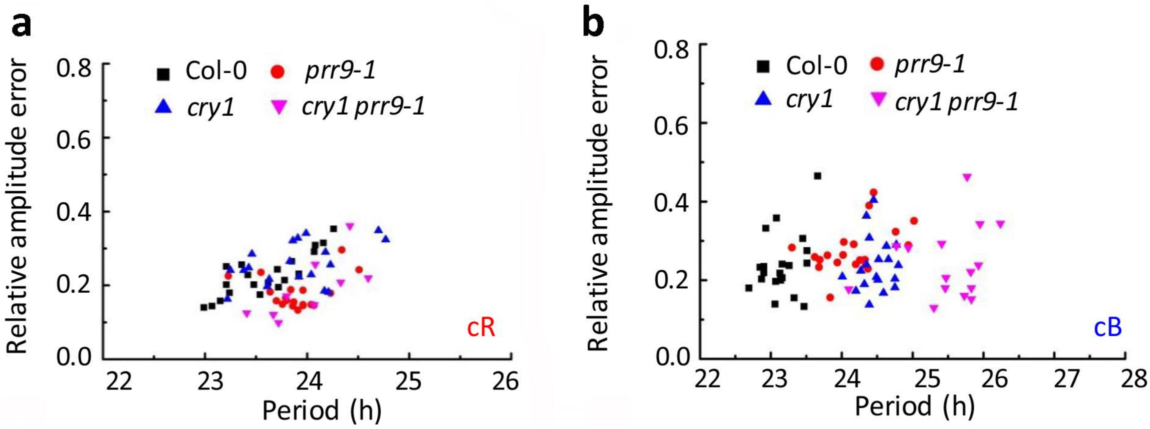

Supplementary Fig. 6: CRY1 and PRR9 act additively in regulating circadian period in constant blue light.

a, b Scatter plot of the estimated circadian period and relative amplitude error of Col-0, *prr9-1*, *cry1* and *cry1 prr9-1* plants in 40 µmol m^{-2} s^{-1} continuous red (n=20, 19, 19, 18 respectively) (a) or blue light (n=20, 19, 20, 18 respectively) (b). The analysis was carried out three biological repeats with similar results.

Supplementary Table 3: Primers used in this assay.

	Primer Sequence (5'-3')
**BiFC assay**	
35S:PRR9-nYFP-F	ATTTACGAACGATAGTTAATTAAATGGGGGAGATTGTGGTTTT
35S:PRR9-nYFP-R	ACTGCCACCTCCTCC ACTAGT TGATTTTGTAGACGCGTCTG
35S:CRY1-cYFP-F	ATTTACGAACGATAG TTAATTAA ATGTCTGGTTCTGTATCTGG
35S:CRY1-cYFP-R	ACTGCCACCTCCTCC ACTAGT CCCGGTTTGTGAAAGCC
35S:CRY2-cYFP-F	ATTTACGAACGATAG TTAATTAAATGAAGATGG ACAAAAAGAC
35S:CRY2-cYFP-R	ACTGCCACCTCCTCC ACTAGT TTTGCAACCATTTTTTC
35S:PPK1-cYFP-F	ATTTACGAACGATAGTTAATTAAATGCCGGAGCTTCGCCGTGG
35S:PPK1-cYFP-R	ACTGCCACCTCCTCCACTAGTAGTAGATACAGTTCGGCCATAG
35S:PPK2-cYFP-F	ATTTACGAACGATAG TTAATTAAATGCCAGAGT TAAGAAGTGG
35S:PPK2-cYFP-R	ACTGCCACCTCCTCCACTAGTAGTGCAAACTGTCCTCCCAAAC
35S:PPK3-cYFP-F	ATTTACGAACGATAG TTAATTAAATGCCAGAGT TAAGAAGTGG
35S:PPK3-cYFP-R	ACTGCCACCTCCTCCACTAGTAGTGCAAACTGTCCGACCATAC
35S:PPK4-cYFP-F	ATTTACGAACGATAG TTAATTAAATGCCTGAGC TGCGTAGCAA
35S:PPK4-cYFP-R	ACTGCCACCTCCTCC ACTAGTAGTTGACACAGTTCGACCATA
**Split Nano LUC assay**	
CsVMV:PRR9-NlucN-R	TAACCTGCCATGGATCCCGGGGTTCATAGGATCTCCTCGAAGAG TCGATAGCCGGTGACTGATTTTGTAGACGCGTCTG
CsVMV:CRY1-NlucC-R	GAGGGTGAAGACCATCCCGGGCCCGGTTTGTGAAAGCC
CsVMV:CRY2-NlucC-R	GAGGGTGAAGACCATCCCGGGTTTGCAACCATTTTTTC
**Co-IP assay**	
CsVMV:PRR9-HA-F	AATTCTGCAGTCGACGGTACC ATGGGGGAGA TTGTGGTTTT
CsVMV:PRR9-HA-R	TAACCTGCCATGGATCCCGGGGTTGATTTTGTAGACGCGTCTG
CsVMV:PRR9-GFP-F	AATTCTGCAGTCGACGGTACC ATGGGGGAGA TTGTGGTTTT
CsVMV:PRR9-GFP-R	TACTCATCCATGGAT CCCGGGC TGATTTTGTAGACGCGTCTG
CsVMV:CRY1-GFP-F	AATTCTGCAGTCGACGGTACC ATGTCTGGTTCTGTATCTGG
CsVMV:CRY1-GFP-R	TACTCATCCATGGAT CCCGGGC CCCGGTTTGTGAAAGCC
CsVMV:CRY2-GFP-F	AATTCTGCAGTCGACGGTACCATGAAGATGGACAAAAAGAC
CsVMV:CRY2-GFP-R	TACTCATCCATGGAT CCCGGGCTTTGCAACCATTTTTTC
CsVMV:CRY2-HA-F	AATTCTGCAGTCGACGGTACCATGAAGATGGACAAAAAGAC
CsVMV:CRY2-HA-R	TAACCTGCCATGGATCCCGGGGTTTTGCAACCATTTTTTC
pENTR2B:CRY2-F	GCTGGGTCTAGATATCTCGAGTCACCCGGTTTGTGAAAGCC
pENTR2B:CRY2-R	GCTGGGTCTAGATATCTCGAGTCATTTGCAACCATTTTTTC
pENTR2B:PHR-R	GCTGGGTCTAGATATCTCGAGTCATCTTGAAATAGCTTTAGC
pENTR2:CCE-F	CAGTCGACTGGATCCGGTACCATGACCCGTGAAGCACAGATA
CsVMV:PRR9N-GFP-R	TACTCATCCATGGAT CCCGGGC ACGCTTGTCAATTCCACCAA
CsVMV:PRR9C-GFP-F	AATTCTGCAGTCGACGGTACCATGCCTGATAGTATTTATAAAGA
CsVMV:PRR9D-HA-F	CCTGATAGTA TTTATAAAGACAAGGCCGTCGTTG CTTTAGAGGA
CsVMV:PRR9D-HA-R	TCCTCTAAAGCAACGACGGCCTTGTCTTTATAAATACTATCAGG
CsVMV:PPK1-GFP-F	AATTCTGCAGTCGACGGTACCATGCCGGAGC TTCGCCGTGG

CsVMV:PPK1-GFP-R	TACTCATCCATGGAT CCCGGGCAGATACAGTTCGGCCATAG
CsVMV:PPK2-GFP-F	AATTCTGCAGTCGACGGTACCATGCCAGAGT TAAGAAGTGG
CsVMV:PPK2-GFP-R	TACTCATCCATGGAT CCCGGGC GCAAACTGTCCTCCCAAAGC
CsVMV:PPK3-GFP-F	AATTCTGCAGTCGACGGTACCATGCCAGAGTTAAGAAGTGG
CsVMV:PPK3-GFP-R	TACTCATCCATGGAT CCCGGGCGCAAACTGTCCGACCATAGC
CsVMV:PPK4-GFP-F	AATTCTGCAGTCGACGGTACCATGCCTGAGCTGCGTAGCAA
CsVMV:PPK4-GFP-R	TACTCATCCATGGATCCCGGGCTGACACAGTTCGACCATA
35S:PPK1-Flag-F	CGAGCTCATGCCGGAGCTTCGCCGTGG
35S:PPK1-Flag-R	CGCGGATCCGTGGTGATGGTGATGATGAGATACAGTTCGGCCATAGC
**Transient expression assay**	
Green-0800-CCA1pro-LUC-F	GTCGACGGTATCGATAAGCTTTCATGCATGGTTAGCTTAGC
Green-0800-CCA1pro-LUC-R	CGCTCTAGAACTAGTGGATCCTCTCCATCACTAAGCTCCTCTAC
UC18-PRR9-3HA-F	ACAGTCGACCAATTGGGTACCATGGGGGAGA TTGTGGTTTT
UC18-PRR9-3HA-R	GTACATAGAGCGGCCCTCGAGTTTTGTAGACGCGTCTG
UC18-CRY2-3HA-F	ACAGTCGACCAATTGGGTACCATGAAGATGGACAAAAAGAC
UC18-CRY2-3HA-R	GTACATAGAGCGGCCCTCGAGTTTGCAACCATTTTTTC
**Site-directed mutation in the primer**	
$PRR9^{S267AS269A}$-F	TGAAAAGATCTTGCGCGGTAGCGTTTGAGAA
$PRR9^{S267AS269A}$-R	TTCTCAAACGCTACCGCGCAAGATCTTTTCA
$PRR9^{S309AT310A}$-F	TAGAGGA GAGTGCTGCA GGTGAGCCAA AGA
$PRR9^{S309AT310A}$-R	TCTTTGGCTCACCTGCAGCACTCTCCTCTA
$PRR9^{T334AT335AS336AS337A}$-F	GAAGCGCCGCAGCGGCCGCCAACCAGGAG
$PRR9^{T334AT335AS336AS337A}$-R	CTCCTGGTTGGCGGCCGCTGCGGCGCTTC
$PRR9^{S365A}$-F	CAGAAGCAAG ATGCACCCAT ACCGGTAGAA
$PRR9^{S365A}$-R	TTCTACCGGTATGGGTGCATCTTGCTTCTG
$PPK1^{K175A}$-F	TTCTCCTTTACTCATCCATGGAGATACAGTTCGGCCATAG
$PPK1^{K175A}$-R	TTCTCCTTTACTCATCCATGGGCAAACTGTCCTCCCAAAGC
**qPCR primers**	
qCCA1-F	CCTTTTACAAACACCGGCTCTT
qCCA1-R	AATCGGGAGGCCAAAATGA
qLHY-F	AAATCAAGATGAGAATTGCTCGGG
qLHY-R	ACTTGTTTCAATGTCGCCACTTACTTTC
qPRR9-F	GTTGAAGAGGAAAGATCGATGCTT
qPRR9-R	CTGCTCTGGTACCGAACCTTTT
qACT2-F	GCTGAGAGATTCAGATGCCCA
qACT2-R	GTGGATTCCAGCAGCTTCCAT
**ChIP-qPCR primers**	
CCA1-A1-F	CAGGTAGTCCCGGAACTCGTGG
CCA1-A1-R	CGGAAATGGAGAAATCTCAGCC
CCA1-A2-F	CTCCATTTCCGTAGCTTCTGG
CCA1-A2-R	CAAACAATAAGAAAGACCATGAC

LHY-A3-F	CACAGCTGGCACCGTACCCAC
LHY-A3-R	CTCAAGTTGCTTCTCTACGAGC
LHY-A4-F	GTTAGAGAGGATTTGAAGCAG
LHY-A4-R	CATCGCTCTCGCTGCTTTG
ChIP-ACT2-F	CGTTTCGCTTTCCTTAGTGTTAGCT
ChIP-ACT2-R	AGCGAACGGATCTAGAGACTCACCTTG
**Pull down assay**	
pMAL2C:PRR9-MBP-F	AGGATTTCAGAATTCGGATCCATGGGGGAGA TTGTGGTTTT
pMAL2C:PRR9-MBP-R	TGCCTGCAGGTCGACTCTAGACTATGATTTTGTAGACGCGTCG
**Primers for PCR scoring**	
ppk2-1-F	CCATGGCAAGGATATCAGGTCTGTTTTT
ppk2-1-R	AATAAAAATGGCATTAAACCGGGGTGAG
ppk3-2-F	ACATCAAAGGTTTGTGCATGGAGAT
ppk3-2-R	TATTCGTAACTGCCTCGAGGAACGCTT
prr9-1-F	GCTGTTTCTGATGGTTTAGC
prr9-1-R	AAACTCGTCTCTTTCATCCA
cry1-LP	AAATCCGCATCAAGAAGTGTG
cry1-RF	TCCACTGGTGCTTCTCAGATC
cry2-1-F	ATGAAGATGGACAAAAAGAC
cry2-1-R	ATACTCTCTTAAACCGATTCCCCTA
LB21	TGGTTCACGTAGTGGGCCATCG

82
