# Supplementary material for: Aschoff’s rule on circadian rhythms orchestrated by blue light sensor CRY2 and clock component PRR9
Source: Nat Commun. 2022 Oct 5;13:5869. doi: 10.1038/s41467-022-33568-3 (PMC9535003; doi:10.1038/s41467-022-33568-3)
Supplement: Supplementary file 3 — Reporting Summary [file 41467_2022_33568_MOESM3_ESM.pdf]

nature portfolio

Corresponding author(s): Lei Wang

Last updated by author(s): 2022-9-20

Reporting Summary

Nature Portfolio wishes to improve the reproducibility of the work that we publish. This form provides structure for consistency and transparency in reporting. For further information on Nature Portfolio policies, see our Editorial Policies and the Editorial Policy Checklist.

Statistics

For all statistical analyses, confirm that the following items are present in the figure legend, table legend, main text, or Methods section.

n/a	Confirmed	
☐	☒	The exact sample size (*n*) for each experimental group/condition, given as a discrete number and unit of measurement
☐	☒	A statement on whether measurements were taken from distinct samples or whether the same sample was measured repeatedly
☐	☒	The statistical test(s) used AND whether they are one- or two-sided<br>*Only common tests should be described solely by name; describe more complex techniques in the Methods section.*
☒	☐	A description of all covariates tested
☐	☒	A description of any assumptions or corrections, such as tests of normality and adjustment for multiple comparisons
☐	☒	A full description of the statistical parameters including central tendency (e.g. means) or other basic estimates (e.g. regression coefficient) AND variation (e.g. standard deviation) or associated estimates of uncertainty (e.g. confidence intervals)
☐	☒	For null hypothesis testing, the test statistic (e.g. *F*, *t*, *r*) with confidence intervals, effect sizes, degrees of freedom and *P* value noted<br>*Give P values as exact values whenever suitable.*
☒	☐	For Bayesian analysis, information on the choice of priors and Markov chain Monte Carlo settings
☒	☐	For hierarchical and complex designs, identification of the appropriate level for tests and full reporting of outcomes
☒	☐	Estimates of effect sizes (e.g. Cohen's *d*, Pearson's *r*), indicating how they were calculated

Our web collection on statistics for biologists contains articles on many of the points above.

Software and code

Policy information about availability of computer code

Data collection	CCD camera (LN/1300-EB/1, Princeton Instruments) was used to capture bioluminescence signals in bioluminescence assay;<br>Olympus FV1000MPE confocal microscope was used to capture images in BiFC assay;<br>CCD camera (Tanon-5200) were used to expose and capture images in western blot, transient expression assay in tobacco system and split Nano-Luciferase complementation assay;<br>Promega GloMax 20/20 was used in Arabidopsis protoplasts transient expression assay;<br>Thermo Scientific Orbitrap Fusion Lumos Tribrid mass spectrometer coupled with chromatography system were used for IP-MS assay;<br>Applied Biosystems™ QuantStudio 3 instrument (Applied Biosystems) was used in qPCR and ChIP-qPCR.
Data analysis	MetaMorph Microscopy Automation and Image Analysis Software were used to analyze bioluminescence signals, circadian period and relative amplitude;<br>Image J was used to analyze band intensity of pictures in western blot and angels of fluence response curves;<br>SPSS software (https://www.ibm.com/products/spss-statistics) was used in one-way ANOVA followed by Fisher's LSD test;<br>Excel 2016 was used in two-side student's t-test, qPCR and ChIP-qPCR data analysis;<br>OriginPro 8.5.7 was used in forming bioluminescence traces, scatter plot and circadian period graph;<br>Adobe Photoshop CS6 was used to cut images from western blot and confocal microscope;<br>Orbitrap Fusion Lumos Tribrid platform, Thermo Scientific™ Proteome Discoverer™ 2.4 software and Uniprot-Arabidopsis thaliana database was used for peptide analysis, data analysis and data search.

For manuscripts utilizing custom algorithms or software that are central to the research but not yet described in published literature, software must be made available to editors and reviewers. We strongly encourage code deposition in a community repository (e.g. GitHub). See the Nature Portfolio guidelines for submitting code & software for further information.

Data

Policy information about availability of data

All manuscripts must include a data availability statement. This statement should provide the following information, where applicable:

- Accession codes, unique identifiers, or web links for publicly available datasets
- A description of any restrictions on data availability
- For clinical datasets or third party data, please ensure that the statement adheres to our policy

The source data for Figs.1-7, Supplementary Figs. 1-6 are provided with this paper as a Source Data file. Other materials of this study are available from the corresponding author upon reasonable request. IP-MS data for PRR9-interacting proteomics are available via PtoteomeXchange with identifier PXD035252.

Human research participants

Policy information about studies involving human research participants and Sex and Gender in Research.

Reporting on sex and gender	*Use the terms sex (biological attribute) and gender (shaped by social and cultural circumstances) carefully in order to avoid confusing both terms. Indicate if findings apply to only one sex or gender; describe whether sex and gender were considered in study design whether sex and/or gender was determined based on self-reporting or assigned and methods used. Provide in the source data disaggregated sex and gender data where this information has been collected, and consent has been obtained for sharing of individual-level data; provide overall numbers in this Reporting Summary. Please state if this information has not been collected. Report sex- and gender-based analyses where performed, justify reasons for lack of sex- and gender-based analysis.*
Population characteristics	*Describe the covariate-relevant population characteristics of the human research participants (e.g. age, genotypic information, past and current diagnosis and treatment categories). If you filled out the behavioural & social sciences study design questions and have nothing to add here, write "See above."*
Recruitment	*Describe how participants were recruited. Outline any potential self-selection bias or other biases that may be present and how these are likely to impact results.*
Ethics oversight	*Identify the organization(s) that approved the study protocol.*

Note that full information on the approval of the study protocol must also be provided in the manuscript.

Field-specific reporting

Please select the one below that is the best fit for your research. If you are not sure, read the appropriate sections before making your selection.

☒ Life sciences ☐ Behavioural & social sciences ☐ Ecological, evolutionary & environmental sciences

For a reference copy of the document with all sections, see nature.com/documents/nr-reporting-summary-flat.pdf

Life sciences study design

All studies must disclose on these points even when the disclosure is negative.

Sample size	No statistical method were used to predetermine sample size. Required experimental sample sizes were estimated based on previous established protocols. The same sizes were adequate as the experimental results were reproducible.
Data exclusions	No data were excluded from analysis
Replication	All experiments were reproduced in three independent biological experiments .
Randomization	Samples were randomly collected
Blinding	Blinding was not possible as the authors who performed the experiments also analyzed the data.

Reporting for specific materials, systems and methods

We require information from authors about some types of materials, experimental systems and methods used in many studies. Here, indicate whether each material, system or method listed is relevant to your study. If you are not sure if a list item applies to your research, read the appropriate section before selecting a response.

Materials & experimental systems

n/a	Involved in the study
☐	☒ Antibodies
☒	☐ Eukaryotic cell lines
☒	☐ Palaeontology and archaeology
☒	☐ Animals and other organisms
☒	☐ Clinical data
☒	☐ Dual use research of concern

Methods

n/a	Involved in the study
☒	☐ ChIP-seq
☒	☐ Flow cytometry
☒	☐ MRI-based neuroimaging

Antibodies

Antibodies used	Anti-HA High Affinity, Roche, Cat# 11867423001;<br>Rabbit polyclonal to GFP, Abcam, Cat#ab6556;<br>Anti-Plant-actin Rabbit Polyclonal Antibody, EASYBIO, Cat#BE0027-100;<br>DYKDDDDK-Tag (3B9) mAb, Abmart, Cat#M20008L;<br>Mouse polyclonal to PRR9, made in Animal Centre, Institute of Genetics and Developmental Biology, Chinese Academy of Science;<br>Anti-Cryptochrome-2 Antibody, PHYTOAB, Cat#PHY1939;<br>Rabbit polyclonal to Histone H3-Nuclear Loading Control and ChIP Grade, Abcam, Cat# ab1791;<br>ChIPAb+™ Acetyl-Histone H3 (Lys9) Serum, Merck-Millipore, Cat# 17-609;<br>Anti-HA tag antibody-ChIP grade, Abcam, Cat# ab9110.
Validation	PRR9 antibody is made in laboratory animal center, Institute of Genetics and Developmental Biology.<br>All other antibodies used in this paper were certified and validated by manufactures.<br>The details about Anti-HA High Affinity is in https://www.casmart.com.cn/product-details/page/243/515071108<br>The details about Rabbit polyclonal to GFP is in https://www.casmart.com.cn/product-details/page/2945/421763290<br>The details about Anti-Plant-actin Rabbit Polyclonal Antibody is in https://www.casmart.com.cn/product-details/page/402/18761929<br>The details about DYDDDDK-Tag (3B9) mAb is in https://www.casmart.com.cn/product-details/page/300028763/442593265<br>The details about Rabbit polyclonal to Histone H3-Nuclear Loading Control and ChIP Grade is in https://www.casmart.com.cn/product-details/page/1155/54647928<br>The details about ChIPAb+™ Acetyl-Histone H3 (Lys9) Serum is in https://www.casmart.com.cn/product-details/page/1282/46532642<br>The details about Anti-HA tag antibody- ChIP Grade is in https://www.casmart.com.cn/product-details/page/243/535057033<br>The details about Anti-Cryptochrome-2 Antibody is in https://www.phytoab.com/cry2%20antibody
